# Using diel movement behavior to infer foraging strategies related to ecological and social factors in elephants

**DOI:** 10.1186/2051-3933-1-13

**Published:** 2013-12-03

**Authors:** Leo Polansky, Iain Douglas-Hamilton, George Wittemyer

**Affiliations:** Department of Fish, Wildlife, and Conservation Biology, Colorado State University, 80523-1474 Ft. Collins, Colorado USA; Save the Elephants, P.O. Box 54667, Nairobi, Kenya; Department of Zoology, University of Oxford, South Parks Road, Oxford, OX1 3PS UK; Graduate Degree Program in Ecology, Colorado State University, 80523-1474, Ft. Collins, Colorado USA

**Keywords:** Fourier analysis, Generalized linear mixed model, Movement ecology, Optimal foraging theory, Savannah, Socio-ecological model, Wavelets

## Abstract

**Background:**

Adaptive movement behaviors allow individuals to respond to fluctuations in resource quality and distribution in order to maintain fitness. Classically, studies of the interaction between ecological conditions and movement behavior have focused on such metrics as travel distance, velocity, home range size or patch occupancy time as the salient metrics of behavior. Driven by the emergence of very regular high frequency data, more recently the importance of interpreting the autocorrelation structure of movement as a behavioral metric has become apparent. Studying movement of a free ranging African savannah elephant population, we evaluated how two movement metrics, diel displacement (DD) and movement predictability (MP - the degree of autocorrelated movement activity at diel time scales), changed in response to variation in resource availability as measured by the Normalized Difference Vegetation Index. We were able to capitalize on long term (multi-year) yet high resolution (hourly) global positioning system tracking datasets, the sample size of which allows robust analysis of complex models. We use optimal foraging theory predictions as a framework to interpret our results, in particular contrasting the behaviors across changes in social rank and resource availability to infer which movement behaviors at diel time scales may be optimal in this highly social species.

**Results:**

Both DD and MP increased with increasing forage availability, irrespective of rank, reflecting increased energy expenditure and movement predictability during time periods of overall high resource availability. However, significant interactions between forage availability and social rank indicated a stronger response in DD, and a weaker response in MP, with increasing social status.

**Conclusions:**

Relative to high ranking individuals, low ranking individuals expended more energy and exhibited less behavioral movement autocorrelation during lower forage availability conditions, likely reflecting sub-optimal movement behavior. Beyond situations of contest competition, rank status appears to influence the extent to which individuals can modify their movement strategies across periods with differing forage availability. Large-scale spatiotemporal resource complexity not only impacts fine scale movement and optimal foraging strategies directly, but likely impacts rates of inter- and intra-specific interactions and competition resulting in socially based movement responses to ecological dynamics.

## Background

Seasonal resource fluctuations dominate many ecological processes including structuring the movements of large herbivores across the landscape [1, 2]. Understanding how fine-scale movement changes in response to different spatial and temporal scales of resource fluctuations offers an approach to evaluate how animals respond to dynamic resource landscapes in order to maximize fitness [2]. Organismal movement is thought to be driven by the interplay between external and internal conditions [3], where optimal movement strategies can vary in relation to the interplay between ecological (external) and physiological (internal) conditions [4]. As such, different optimal movement strategies may be elicited depending on the aims of the organism (e.g. focused on energy conservation vs. forage acquisition and energy maximization, etc.) providing opposing predictions of optimal foraging under different ecological regimes.

In addition to ecological and physiological drivers of movement behavior, social factors may also determine space use and behavior in social animal systems [5, 6]. In particular, higher social status can confer benefits in situations where resource distributions allow contest competition, while rank related differences are not expected in scramble competition situations [7]. By contrasting social rank related differences in fine scale movements, it may be possible to better infer the relative constraints and drivers of optimal movement behavior as a function of ecosystem variables. To date, few studies have quantified how the interaction between social status and ecosystem properties structures differences in individual movement behaviors.

Given location data at sufficiently fine scales and with sufficient regularity, at least two movement descriptors can be used to provide data driven insights into movement strategies by foraging herbivores. The first descriptor of movement behavior we study here is total diel displacement (DD), defined as the daily sum of net displacements throughout the day. DD is a proxy for energy expenditure and by extension the foraging strategy employed by an organism. Classical optimal foraging theory (OFT) explicates how movement to a food patch is related to the costs of traveling, the patch quality at its current location, and the average quality of patches throughout the landscape [8]. Conceptually, this has been interpreted as a driver for greater movement (increased DD) as resources decline (less time spent in a patch). But actual application has been limited by difficulties in definiting patch boundaries and quality in real landscapes. Empirical data from several large African herbivore species has supported the observation that increased movement is associated with decreased resource availability [1, 4]. In contrast, recent work [2] explicates how wet season, resource quality and availability is related to increased fine scale heterogeneity relative to uniformly poor dry season resources, which following the prediction of optimal foraging theory [8] can elicit increased movement frequencies (higher DD) as an energy maximization strategy.

The second movement descriptor we studied is the amount of movement autocorrelation in spatial displacement at diel time scales. Elevated levels of activity that are periodically elevated at diel frequencies related to ambient conditions such as light and temperature in many organisms have been recognized for decades (e.g. [9–11]), the drivers of which are often thought to be physiological. In a movement context, periodic activity has been studied as a behavioral signal in large free ranging wildlife species (e.g. [4, 12–15]). Here, we define movement predictability (MP) of fine scale behavior as the proportion of daily movement activity that is significantly periodic with frequencies of at least 1 cycle/day. Under the assumption that autocorrelation in fine scale movement activity (highly predictable variation across the day) reflects preferred movement timing by an organism, analysis of MP gives insight into optimal movement behavior [16]. While MP and DD are not necessarily independent (conditions leading to changes in one could lead to changes in the other), in the movement ecology framework of Nathan et al. [3], MP can be thought of as a measure in the regularity of ‘when’ to move and compliments the DD proxy which is often analyzed in research designed to infer ‘why’ an individual moves.

Here we assess the movement strategies of free ranging African savannah elephants (*Loxodonta Africana*) in the Samburu and Buffalo Springs National Reserves Complex using these two movement descriptors. Prior studies of savannah elephants have revealed complex patterns related to both ecosystem changes [17, 18] and differences in social rank [6, 15, 19], though analyses were limited to relatively short periods (single season). We analyze multi-year fine scale movement (hourly locations over multiple dry-wet seasons per individual) of individuals with known social status, leveraging rank based differences to interpret optimal strategies for given ecological conditions. As outlined previously, theory provides the foundation for opposing predictions, to which we apply our rank based comparative framework to interpret preferred behaviors as those conducted by dominant individuals. Under this framework, we test the following optimal foraging theory based predictions regarding movement and dynamics in forage availability:

*Diel displacement (DD)*: (i) If costs associated with increased movement are offset by increased energy accumulation, optimal foraging theory [8] predicts DD will increase with decreasing levels of forage availability per unit area as individuals must move further to obtain equivalent levels of energy. (ii) Alternatively, if the costs of taxis in dry season conditions outweigh any expected energetic gains achieved by increased movement, individuals may opt to minimize DD during dry season conditions in order to conserve energy. Equivalently, under the assumption that wet season conditions associated with high forage availability also produce increased fine scale heterogeneity in resource quality and availability (that is, dry season conditions are associated with uniformly poor resources), increased movement frequencies, and thus higher DD, may demonstrate optimal foraging behavior related to an energy maximization strategy in the wet season.

*Movement predictability (MP)*: (i) The dry season impacts of declining forage availability and constricted water sources could increase MP given the increased pressure to time movement in an energy conserving strategy. (ii) Alternatively, if the probability of finding high value food patches declines to where individuals either resist moving or must continuously move, then MP would decline with declining forage availability. As outlined in previous work [15], declines in MP could also reflect increased rates of inter- and intra-specific competition that erode the ability to follow ideal timing in movement [20].

## Results

### Diel displacement model

**Figure 1:**
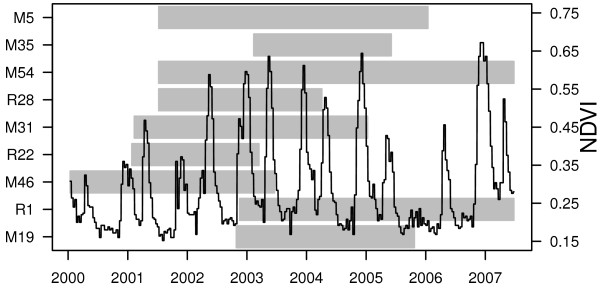
**Data overview.** The grey bars delineate the temporal extent of the tracking dataset for each individual, and ecosystem productivity as measured by NDVI is shown by the black line. Days with less than 20 location fixes were excluded from the analyses- see Table 1.

**Figure 2:**
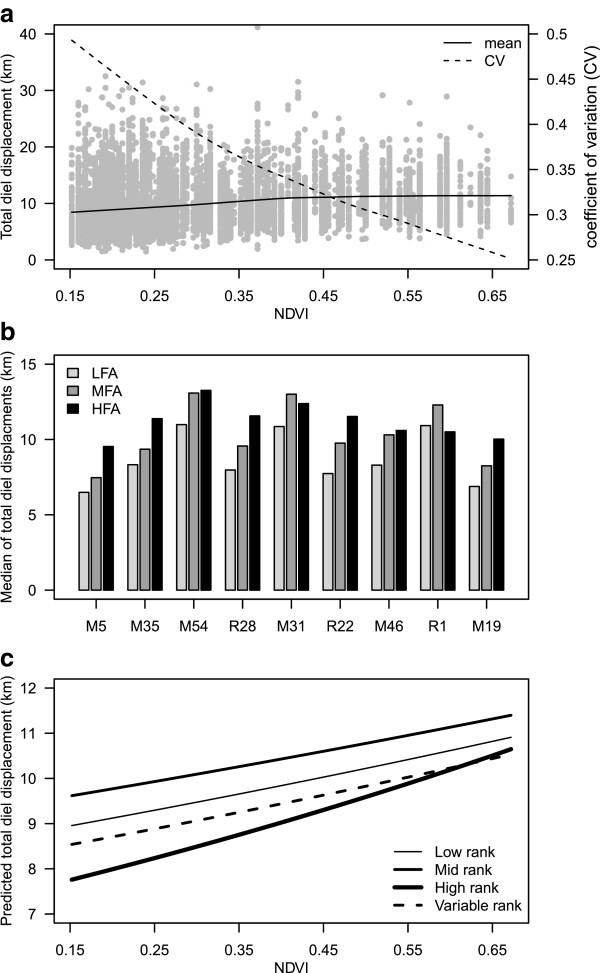
**Total diel displacement vs. NDVI. (a)** Daily travel distance (km) vs. NDVI values for all individuals (grey points). Loess smoothers using linear regression show trends of the mean (solid line) and coefficient of variation (dashed line) as a function of NDVI. **(b)** Median daily travel distances for individuals during low (LFA), medium (MFA), and high (HFA) forage availability seasons. **(c)** Model predicted DD values by rank at the median values of the lagged DD covariate terms by social rank.

Individuals averaged 910 days of tracking, of which approximately 90% was used in this analysis (summarized in Table 1 and Figure 1). Summary statistics across all individuals (Figure 2a), or by each individual separately (Figure 2b), indicate a positive association between DD and forage availability as measured by Normalized Difference Vegetation Index (NDVI) data aggregated for the ecosystem. Averaging across all individuals, mean DD increased with higher NDVI values while the coefficient of variation (CV) decreased (Figure 2a). Median DD averaged across individuals increased monotonically across three seasonal quality categories (low, medium and high forage availability or LFA, MFA, and HFA), with LFA, MFA, and HFA seasons values of 8.72 km, 10.34 km, and 11.20 km, respectfully. DD values were occasionally greater than 20 km, with a maximum of 39.15 km in 24 hours (Figure 2a), but were typically less than 20 km (0.95 quantile = 18.35 km for all individuals over all days). However, the individual response to NDVI was variable with not all individuals demonstrating monotonic increases in median DD with increases in NDVI (Figure 2b).

**Table 1:** **Data summary for all female elephants with >2 years of tracking data from Save the Elephants’s tracking program**

Individual	Relative rank	Start day	End day	Number of tracking days
M5	High	9-Jul-2001	14-Jan-2006	1371 (1273)
M35	High	11-Feb-2003	3-Jun-2005	775 (671)
M54	High	8-Jul-2001	25-Jun-2007	1447 (1310)
R28	High	9-Jul-2001	4-Apr-2004	527 (519)
M31	Mid	9-Feb-2001	9-Jan-2005	703 (689)
R22	Mid	25-Jan-2001	16-Mar-2003	739 (729)
M46	Low	13-Jan-2000	19-Jun-2003	544 (518)
R1	Low	17-Nov-2002	25-Jun-2007	1230 (1034)
M19	Variable*	10-Oct-2002	23-Oct-2005	854 (592)

The tracking day counts show days with at least 20 hours of successful location downloads and are the data used in the binning and smoothing spline based analyses, and counts shown in the parentheses are sample sizes for unique triplets of three consecutive days with at least 20 hours of successful location downloads used in the regression models. The relative ranks reflect that of the collared elephant’s matriarch. *M19’s matriarch died midway through the study.

We built generalized linear mixed effect models (GLMM) directly relating NDVI and social status to properties of movement, while including a stochastic structure that accommodates individual heterogeneity and terms for endogenous autocorrelation, without which residuals showed high autocorrelation. The GLMM analysis indicated a statistically significant and positive affect of NDVI on DD and a significant interaction between social rank and NDVI (Table 2). Fixed effect slope estimated by rank (obtained by adding the NDVI + NDVI:rank term coefficients in Table 2) are 0.38, 0.33, 0.40, and 0.61 for the low, medium, variable, and high rank factors. Comparing these slopes indicates that lower ranking individuals show relatively smaller changes in the DD response to NDVI compared with high ranking individuals (61% increase in the high ranking slope estimate over low-ranking individuals). Figure 2c illustrates these rank related differences with the first and second lagged covariate terms *n*_*i*-1*,j*_ and *n*_*i*-2*,j*_ set at their medians across all individuals (as necessitated to graphically illustrate predictions which include autocorrelation).

**Table 2:** **Parameter statistics for the fixed effects terms of the diel displacement (DD) model**

Parameter	Estimate	SE	t-value	***χ*** ^2^	df	P-value
Intercept	1.04	0.05	22.18	2097.17†	9	<0.01
NDVI	0.38	0.07	5.6	*		
Rank medium	0.08	0.05	1.46	*		
Rank variable	-0.05	0.07	-0.75	*		
Rank high	-0.18	0.05	-3.83	*		
NDVI: Rank medium	-0.05	0.1	-0.54	*		
NDVI: Rank variable	0.02	0.12	0.19	*		
NDVI: Rank high	0.23	0.08	2.82	15.46††	3	<0.01
1 day lag term	0.19	0.01	17.26	589.36†††	1	<0.01
2 day lag term	0.31	0.01	27.59	722.45††††	1	<0.01

The overall reference intercept parameter corresponds to the low ranking individuals, to which rank parameter terms are added to obtain rank specific intercepts. *χ* ^2^ values show the likelihood ratio test, with degrees of freedom (df) and associated P-value. :- indicates interaction term. *- Significance test not meaningful for single terms involved in significant interactions. † - Significance test of the full model against a null model with only an individual random effect term. †† - Test of the significance of the interaction term against the reduced model that includes both rank and NDVI but not their interaction. ††† - Test of whether the 1 day lag term is needed against a model with no lag terms. †††† - Test of whether a second lag term is needed.

### Movement predictability model

**Figure 3:**
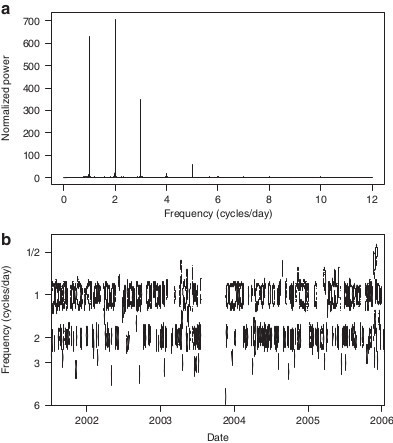
**Fourier and wavelet illustrations.** The Fourier and wavelet power spectra for M5, the basic features of which are typical of all the individuals studied here, illustrate how movement predictability (MP) is revealed through intermittent movement autocorrelation. Panel **(a)** shows the Fourier spectrum computed using a Tukey (bell cosine) taper and smoothed with a modified Danielle smoother over the immediately adjacent frequency locations. For easy comparison against a random walk process that corresponds to movement with no MP, the power is normalized so that a white noise signal with variance equal to the variance in the observed net displacement time series has the constant power spectra of one across all frequencies. Spikes in power indicate significant periodic activity at 1, 2 and 3 cycles per day. Panel **(b)** shows contour plots of significant regions in the wavelet power spectrum, which allows time-localized determination of when MP is present, and shows that MP is not constant through time. Areas within closed curves (thick black lines) indicate movement that is significantly periodic.

**Figure 4:**
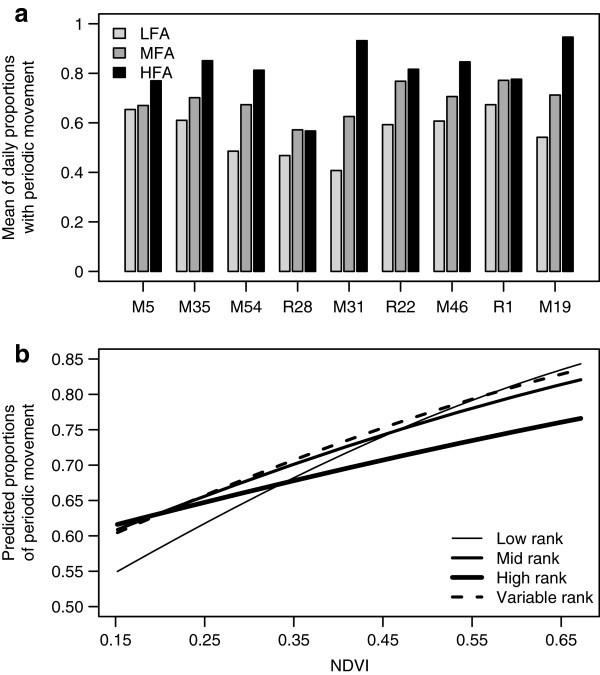
**Daily movement predictability vs. NDVI. (a)** Mean proportion of time with significant movement autocorrelation during low (LFA), medium (MFA), and high (HFA) forage availability seasons for each individual. **(b)** Model predicted daily MP by rank at the mean values of the lagged daily MP covariate terms by social rank.

Figure 3 provides an illustrative example of a wavelet transform of the step lengths for an individual, the basis for which to extract the measure of MP at diel scales. The mean daily MP statistic averaged across all individuals is 0.56, 0.69, 0.81, for the LFA, MFA, and HFA forage indices, respectfully. At the individual level, all individuals showed positive increases in mean daily MP with increases in NDVI by season (Figure 4). Variation in the proportion of time with periodic movement across forage availability categories appeared to be high both within individuals (e.g. M31 ranges from ~40% during LFA times to ~90% during the HFA times) and across individuals (e.g. the highest proportion of time with autocorrelated movement for R28 is lower than the lowest proportion for many other individuals).

As with the DD analysis, the GLMM analysis of daily MP indicated a statistically significant positive affect of NDVI on daily MP and a significant interaction between social rank and NDVI (Table 3). Fixed effect slope estimates on a linear scale by rank are 2.85, 2.07, 2.31, and 1.37 for the low, medium, variable, and high rank factors. Comparing these slopes indicated that lower ranking individuals show relatively larger changes in the MP response to NDVI compared with high ranking individuals (52% decrease in the high ranking slope estimate over low-ranking individuals). Figure 4b illustrates these rank related differences with the first and second lagged covariate terms *n*_*i*-1*,j*_ and *n*_*i*-2*,j*_ set at their means across all individuals.

**Table 3:** **Parameter statistics for the fixed effects terms of the daily movement predictability (MP) model**

Parameter	Estimate	SE	z-value	***χ*** ^2^	df	P-value
Intercept	-2.08	0.13	-16.05	73029.07†	9	<0.01
NDVI	2.85	0.12	23.91	*		
Rank medium	0.36	0.18	1.97	*		
Rank variable	0.31	0.23	1.36	*		
Rank high	0.5	0.16	3.15	*		
NDVI: Rank medium	-0.78	0.17	-4.63	*		
NDVI: Rank variable	-0.54	0.22	-2.48	*		
NDVI: Rank high	-1.48	0.14	-10.56	123.02††	3	<0.01
1 day lag term	0.17	0	184.68	64042.21†††	1	<0.01
2 day lag term	-0.04	0	-50.35	2823.9††††	1	<0.01

The overall reference intercept parameter corresponds to the low ranking individuals, to which rank parameter terms are added to obtain rank specific intercepts. *χ* ^2^ values show the likelihood ratio test, with degrees of freedom (df) and associated P-value. :- indicates interaction term. *- Significance test not meaningful for single terms involved in significant interactions. † - Significance test of the full model against a null model with only an individual random effect term. †† - Test of the significance of the interaction term against the reduced model that includes both rank and NDVI but not their interaction. ††† - Test of whether only the 1 day lag term is needed. †††† - Test of whether the second lag term is needed.

## Discussion

### Insights to movement strategies from long term data

Large herbivores are thought to respond to large-scale ecological processes given physiological constraints [2, 21]. As such, understanding movement behavioral changes in response to large-scale ecological changes is critical for understanding the movement ecology of large mammals. While investigating the influence of dynamic resource environments on animal movement has been a long standing aim in the ecological sciences [22, 23], studies of fine-scale movement typically have focused on shorter time frames (i.e. a single season or year), while studies of longer time-scale behaviors have focused on coarser metrics of movements (i.e. migration events). Here we take advantage of modern statistical tools and empirical data to analyze two fine scale metrics of movement behavior in relation to large-scale ecosystem changes and sociality. This allows broader inspection of the influence of ecological fluctuations on movement behavior and the behavioral mechanisms adopted by species to cope with the ecological constraints they face.

We found that all individuals in this population increased their DD with increased forage availability in the study system and that social rank status also significantly interacted with NDVI to influence the rate of these changes: increases in rank, in general, led to larger changes in DD and smaller changes in MP as forage availability increased. Under OFT, this suggests higher ranking individuals are able to more dramatically switch their foraging strategies from energy maximization to energy conservation as resource availability declines. The observation that DD becomes ‘noisier’ (increased CV) in dry season conditions suggests increased stochasticity in the drivers of overall energy expenditure. Note that because of significant individual variation, higher ranking individuals may still at times move more during dry season conditions than lower ranking individuals (e.g. individual M54 vs. M19 in Figure 2b), so that increased variability (as measured by the coefficient of variation) in DD is likely not simply an arithmetic consequence of declining mean DD. Fine scale forage data would be needed to determine whether differences across individuals are driven by regional differentiation in forage availability and distribution, or if other constraints (e.g. group size) are significantly determining overall magnitudes of DD values.

Likewise, all individuals in this population increased their MP as forage availability in the study system increased, but in contrast to DD, increases in rank led in general to smaller changes in MP as forage availability increased. The greater MP during wet season conditions overall suggest that individuals are more easily and directly able to respond to physiological constraints like temperature when forage is not as constraining. During dry season conditions, we speculate that increased rates of conspecific interactions and human interactions are likely to play nontrivial roles in explaining changes in MP by elephants in this open study system. Movement predictability has been shown to decline when elephants were in human dominated areas of this study system [20]. As the primary predator of elephants, human interactions potentially disrupt preferred movement by individuals, scrambling the sequence and times during which they exhibit relatively active or inactive movement (leading to a decline in MP), and forcing more opportunistic foraging and relocation strategies. Human-elephant interactions in the study system tend to increase during the dry season, when both species focus on limited resources such as water [20] and illegal killing of elephants significantly impacts the study population [24]. As such, it is possible human mediated scrambling may drive seasonal differences in MP rather than an inability to adjust behavior to seasonal ecological variation. Further, lower ranked groups tended to spend more time outside the protected areas in human dominated areas of the study system [6], potentially explaining the rank related differences observed in MP.

Taken together, these two analyses suggest that all individuals share a common response to changes in the environment and that the underlying causes of ‘why’ and ‘when’ to move are at least to some extent shared. The need to incorporate autocorrelation terms in both models indicated that we were missing other important shared explanatory variables determining both DD and MP, particularly those operating at scales of approximately 2 days. We speculate that some combination of cumulative physiological requirements and memory of salient resource locations [20, 25] are additional variables that strongly determine these two movement descriptors.

### Study design

Despite having well over 1000 observations (each involving at least 20 incremental movement locations per day) per rank status (Table 1) spread over a wide range of NDVI values (Figure 2a), the total number of individuals available for this study are few. Subsequent lack of confidence in both biological and statistical inference must be discussed. From a biological perspective, the resident elephant population of the Samburu and Buffalo Springs National Reserves Complex currently stands at about 550 [24], and it is easy to imagine that 2–3 individuals at a dominance level may not be representative of the entire subpopulation. We note that the individual data studied here is in many ways better thought of as representing group movement because individuals within groups are highly correlated in both social and ecological dimensions [19], thereby meaning these individuals represent ~80 elephants or over 15% of the resident population. Whether there is bias in our findings related to the particular individuals tracked in this study or if individual level variability would remove any of the fixed effect signals we found is beyond the scope of this data to address.

From a statistical perspective, we note for the fixed effect terms the sample size is fairly large for each level of the categorical predictor rank (>>1000) and spreads fairly evenly across the domain of values of the continuous predictor variable NDVI (Figure 2a), and that estimates of random effect variance is not a primary focus here. The random effects were included to avoid pseudo-replication while including all individuals in a single analysis, necessary for making population level inference irrespective of the confidence of associated estimates. Given enough individuals per social rank category, more complicated random effects models than used here could be of interest. For example, using nested levels of grouping factors would allow quantifying variability across individuals within rank status to test whether individual variability is higher within lower ranked than higher ranked individuals as might indicate greater differentiation in movement strategies.

## Conclusions

Cumulative daily travel distances suggests that optimal movement involves a switch from energy maximization to energy conservation as forage resources decline, and that this is coordinated with less predictability in the timing of movement activity as measured by autocorrelation. While movement responses to changes in resource availability were qualitatively similar across all individuals irrespective of social rank, our analyses found that the capacity to modify movement in response to changes in forage availability depended on social rank status. Lower ranking individuals could not decrease their daily travel distances in response to worsening forage conditions as much as higher ranking individuals, but in contrast showed more marked declines in the predictability of their movements. We interpret this as indicating that decreasing movement rates in association with productivity declines is optimal in this system. Following the same logic, maintaining strong periodicity in movement behavior is also optimal. If these interpretations are accurate, the benefits of rank appear to be realized across a wide range of ecological conditions, beyond the periods when resource competition is most prevalent [26]. The importance of individual characteristics such as rank status found here also reinforces need for caution in uniformly applying movement rules and phenomenological models across individuals in theoretical behavioral analyses [27], especially in systems with complex socioecological underpinnings.

Application of optimal foraging theory across in situ systems has been hindered by an inability to account for all critical variables and their interactions (e.g. predation or forage quality, quantity and distribution). In particular, interpreting the optimality of different movement strategies from movement data alone can be difficult when detailed energetic balance (expenditure versus acquisition) and fitness data are lacking, as is common in many studies of free ranging wildlife. Leveraging social based differences provides an avenue to resolve these intractable issues, as evidenced here. Fundamental principles developed through these theories are useful for framing behavioral research [8, 28]. Expanding the scope of movement behavior analyses to include social as well as ecological drivers may be critical for advancing understanding about optimal foraging in other wildlife systems with complex social structuring.

## Methods

### Data collection

Elephants in this study were collared in the region demarcated by 0.3-0.8° N, 37-38° E along the Ewaso N’giro River. Movement data were collected using global positioning system (GPS) collars fitted on nine elephants of distinct family groups as identified in [29]. GPS collars were fitted by a Kenya Wildlife Service (KWS) veterinarian following the protocol established by KWS. The GPS devices recorded a spatial coordinate each hour, from which we computed hourly net displacement ***S***^*N*^ as the great-circle distance between sequential locations using a spherical earth approximation with radius of 6371 km to obtain diel displacement (DD) values as the sum of the hourly displacements over each day.

The rank (calculated from agonistic interactions among the population) of the collared individual was inferred as that of the most dominant individual of the family group [30] where individual ranks were calculated from 419 agonistic interactions external to the individuals’ social group (each individual averaged interactions with 3.8 ± 0.42 individuals outside her family units). Ranks were established using a likely rank order approach [31] and verified using directional consistency metrics [32, 33]. Group sizes among the collared families varied over the study period, and were not significantly correlated with rank [30]; Pearson’s r = 0.434, p-value = 0.282 between rank and maximum group size during the tracking period).

To extract the most information while retaining a high degree of confidence in our estimates of movement, and to facilitate calculation of Fourier and wavelet coefficients using standard algorithms (i.e. fast Fourier transforms), we estimated locations for failed GPS downloads using the points of a linear interpolation between the successful GPS fixes closest in time. All days with less than 20 hours of successful fixes were subsequently removed from analyses.

Rainfall in this semi-arid ecosystem averages approximately 350 mm per year, predominately falling during two rainy seasons generally taking place in April/May and November/December, and is highly stochastic within and between seasons. NDVI is superior to rainfall as a proxy for forage conditions [34] and characterizes regional changes in forage abundance and quality that strongly shape elephant behavior and demography [6, 26]. For analysis across ecological strata, We assigned each day into one of three categories, low forage availability season (LFA), medium forage availability (MFA), and high forage availability (HFA), using three equally wide bins covering the range of observed NDVI values across all movement data.

### Quantifying movement predictability

Within day movement predictability (MP) was quantified using wavelet analysis of the hourly net displacement time series **S**^*N*^ for each individual separately. Wavelet analysis [35] of times series data is similar to Fourier analysis, but identifies a time localized measure of periodicity within the time series **S**^*N*^ to produce a two dimensional array of numbers called the wavelet power spectrum (WPS). The entries in the two dimensional WPS array correspond to the power of the fit between locally periodic functions of different frequencies along one dimension with the time index of **S**^*N*^ in the other, and have been shown to accurately quantify nonrandom, time localized oscillations in wildlife movement activity [14]. Critically, wavelet analyses provide estimates of MP independent of ecological measures. Because the elephants studied here show intermittence between movement modes within each day [15, 19], autocorrelation in movement corresponds to regular circadian patterns of movement mode switching, whereas noisy, uncorrelated movement reflects unpredictable and irregular movement mode changes.

Quantifying significant autocorrelation in **S**^*N*^ at diel frequencies, and thereby identification of MP, was obtained following the significance testing procedures developed by [36, 37] based on 1000 bootstrapped WPS from white noise signals with variance set at the variance of **S**^*N*^. To emphasize detection of periodicity changes rather than changing variance, we normalized the **S**^*N*^ values to lie between 0 and 1 for each day separately. Figure 3 provides an illustrative example. The movement activity spectral signatures of the individuals here are similar to those shown in [15] and [19], which show both strong periodic cycling at 1, 2 and 3 cycles/day, and temporal variability in this cycling. Each time step in the **S**^*N*^ was classified as autocorrelated if any of the WPS values at 1, 2 or 3 cycles/day at that time step was significantly different from white noise and classified random otherwise. The MP is a daily proportion value corresponding to the proportion of hours in each day with significant autocorrelation, and usually is either 0 or 1 (that is, individuals are usually showing strong circadian activity during a day or no circadian activity).

### Regression models

We used a generalized linear mixed model (GLMM) framework [38] to model DD and MP. This framework accommodates random variation across individuals and allows inclusion of any needed control variables while testing for the main effects of interest, the interaction between NDVI and social rank. Fixed effect terms included NDVI, social rank, and the interaction between rank and NDVI, as well as two days of time lags of the response variable to accommodate endogenous autocorrelation. The need to include temporal autocorrelation was evident based on model diagnostics and in other preliminary models of each individual separately. Random intercepts for each individual were included as random effects terms, and control for individual variation and repeated measures. Random intercepts and slopes were highly correlated, so we did not include a random slopes term (see Discussion and examples in [39] and http://lme4.r-forge.r-project.org/lMMwR/). The full models are symbolically expressed as:1

where *n*_*i,j*_ is the response variable of individual *j* on day *i*, *b*_*j*_ are the unobserved random intercepts, and *ϵ*_*i,j*_ are the residuals. For the DD models, *g* is the identity link function, and for the MP model *g* is the logit link function. To make sure that variance heterogeneity did not influence results, we also ran all models using log_e_ transforms of the DD data but found qualitatively identical results; box plots of residuals by individual in all models also indicated fairly sound agreement with model assumptions.

We arrived at the final model structure after first investigating models for each individual separately using generalized linear models. In these preliminary modeling exercises, we found that including an endogenous autocorrelation structure as done here satisfactorily removed residual autocorrelation, while indicating good agreement between model assumptions and data using standard model diagnostic plots of residuals. As a slight but interesting aside, in these preliminary modeling exercises we found the appropriateness of the endogenous autocorrelation structure to be in contrast to modeling autocorrelation exogenously (arising for example from correlated environmental variates) by specifying a non-diagonal residual covariance matrix, which points to a stronger influence of endogenous over exogenous autocorrelation in elephant movement.

GLMM models were fit in the R environment [40] using the lmer function from the lme4 package [39] based on maximum likelihood estimation. Data were obtained from the subset of the overall tracking data with complete days for which at least three consecutive complete tracking days were obtained (Table 1) so that the autocorrelation terms could be included. Model term inference and selection for random effects models is an active area of statistical research [38]. Here we report the estimate, standard error, and t-values or z-values, and test significance using a likelihood ratio test with the anova function (see the 2011 updated supporting information for [38]).

To complement the model based analyses, we performed several preliminary statistical analyses that ignored temporal lagged effects, but provide an easy overview of the data trends and heterogeneity ultimately confirmed using the more robust modeling framework. Specifically, we estimated trends in the means and coefficient of variation (CV) in DD as a function of NDVI for all individuals simultaneously using linear regression smoothing splines (pgs. 228–232 in [41]) fit with the loess function in the R environment [40]. Initial analyses are intended to provide summary descriptors of movement without model abstractions. Median values are chosen for presenting binned descriptions of the DD behavior to diminish the influence of very large values associated with occasional migration events, which are not the focus of these analyses. Bar plots are given to provide an overview of trends, but rather than error bars we rely on the mixed effect regression models to quantify differences and uncertainty; these models allow for robust incorporation of individual level variability and do not require ad hoc binning assignments.

## Availability of supporting data

Contact I. Douglas-Hamilton and G. Wittemyer.

## Abbreviations

CV: Coefficient of variation
DD: Diel displacement
GLMM: Generalized linear mixed model
KWS: Kenya wildlife service
MP: Movement predictability
NDVI: Normalized difference vegetation index.
